# R-RAS2 overexpression in tumors of the human central nervous system

**DOI:** 10.1186/1476-4598-12-127

**Published:** 2013-10-23

**Authors:** Sylvia Gutierrez-Erlandsson, Pedro Herrero-Vidal, Marcos Fernandez-Alfara, Susana Hernandez-Garcia, Sandra Gonzalo-Flores, Alberto Mudarra-Rubio, Manuel Fresno, Beatriz Cubelos

**Affiliations:** 1 Centro Nacional de Biotecnología, Consejo Superior de Investigaciones Científicas, Universidad Autónoma de Madrid, Madrid 28049, Spain; 2 Centro de Biología Molecular Severo Ochoa, Departamento de Biología Molecular, Universidad Autónoma de Madrid, Madrid 28049, Spain

**Keywords:** RAS family proteins, R-RAS2, CNS tumors, TC21

## Abstract

Malignant tumors of the central nervous system (CNS) are the 10^th^ most frequent cause of cancer mortality. Despite the strong malignancy of some such tumors, oncogenic mutations are rarely found in classic members of the *RAS* family of small GTPases. This raises the question as to whether other *RAS* family members may be affected in CNS tumors, excessively activating *RAS* pathways. The *RAS*-related subfamily of GTPases is that which is most closely related to classical Ras and it currently contains 3 members: *RRAS*, *RRAS2* and *RRAS3*. While R-RAS and R-RAS2 are expressed ubiquitously, R-RAS3 expression is restricted to the CNS. Significantly, both wild type and mutated *RRAS2* (also known as TC21) are overexpressed in human carcinomas of the oral cavity, esophagus, stomach, skin and breast, as well as in lymphomas. Hence, we analyzed the expression of R-RAS2 mRNA and protein in a wide variety of human CNS tumors and we found the R-RAS2 protein to be overexpressed in all of the 90 CNS cancer samples studied, including glioblastomas, astrocytomas and oligodendrogliomas. However, R-Ras2 was more strongly expressed in low grade (World Health Organization grades I-II) rather than high grade (grades III-IV) tumors, suggesting that R-RAS2 is overexpressed in the early stages of malignancy. Indeed, R-RAS2 overexpression was evident in pre-malignant hyperplasias, both at the mRNA and protein levels. Nevertheless, such dramatic changes in expression were not evident for the other two subfamily members, which implies that RRAS2 is the main factor triggering neural transformation.

## Background

The *RAS* family of small GTPases is comprised of 36 genes that encode 37 proteins. It is classified into 21 subfamilies, of which only genes in the classic *RAS* subfamilies (*HRAS*, *NRAS* and *KRAS*) and *RRAS2* have been seen to carry mutations in human cancers [1]. *RRAS2* (also known as TC21) is a member of the *RAS-Related* (*RRAS*) subfamily, which also includes *RRAS* (referred to henceforth as *RRAS1*) and *RRAS3* (also known as *MRAS*) [2]. Of the 3 members of this subfamily, R-RAS2 is the most similar to classic RAS (55-60% protein identity) [3] and this gene has been shown to be mutated in a leiomyosarcoma cell line, a breast cancer cell line, as well as in ovarian carcinoma [4-7]. However, overexpression of the wild type form of R-RAS2 has also been frequently detected in carcinomas of the breast, skin, esophagus, stomach and oral cavity. Furthermore, *RRAS2* is the only RAS family member besides classical RAS that can transform NIH-3 T3 cells. Hence, there is strong evidence that *RRAS2* may be involved in the transformation related to different types of cancer [8-11].

The role of R-RAS2 in normal tissue has been studied in *RRas2*^*−/−*^ mice, which are lymphopenic due to defects in the proliferation and survival of both T and B cells [12]. Interestingly, while mice heterozygous for the null mutation (*RRas2*^*+/−*^) express 50% of the protein found in wild type mice, they are phenotypically more similar to the null knockout mice than to wild type controls [13]. In conjunction with the frequently observed overexpression of wild type R-RAS2 in human cancer, these observations suggest that the expression of the R-RAS2 must be tightly regulated during normal tissue development and homeostasis.

The switch I and II effector domains of R-RAS2 are identical to those of classical RAS [3], indicating that R-RAS2 may also act in the pathways activated by classical RAS [14]. However, while classic RAS exerts its pro-proliferative activity via the activation of the Raf-ERK pathway of MAP kinases, R-RAS2 appears to activate this pathway poorly as it does not recruit Raf1 [15], although it is a strong activator of the PI3K pathway [16,17].

In the USA, malignant tumors are diagnosed in the central nervous system (CNS) of 6.6 out of every 100,000 individuals each year, affecting mostly children and the elderly. In developed countries they are associated with a mortality of 2.7 per 100,000 men and women each year, the 10^th^ most frequent cause of mortality due to cancer [18]. Although some of these tumors are very malignant, mutations in classic RAS members are rare, even though the activity of RAS effectors like PI3K may be enhanced [19-21]. Indeed, since mutations in classic RAS genes, specifically in NRAS [22], were only detected in 2 out of 94 glioblastomas studied, we investigated whether the expression of other RAS family members may be altered in human CNS tumors. Accordingly, we studied the expression of R-Ras2 mRNA and protein in a wide variety of human CNS tumors. R-RAS2 was overexpressed in samples of 288 different human CNS cancers, including glioblastomas, astrocytomas and oligodendrogliomas. However, R-Ras2 was more strongly expressed in low-grade (grades I-II) tumors rather than in high grade tumors (III-IV), suggesting that R-RAS2 is overexpressed in the early stages of malignancy.

## Methods

### Western blotting

Brain tissue samples were homogenized in 1 ml of lysis buffer (50 mM Tris [pH 8], 150 mM NaCl, 1% NP40 and a protease inhibitor cocktail [Sigma-Aldrich, USA]), incubated on ice for 45 minutes and centrifuged at 11,000 rpm. The protein concentration of the lysate was measured by spectrophotometry, and the proteins were then resolved on 15% SDS-PAGE gels (80 μg/well) and transferred to a nitrocellulose membrane. The membrane was probed overnight at 4°C with a specific rabbit antiserum against R-Ras2 (1:1000) purified with a GST-R-Ras2 fusion protein [12]. The *GST-RRAS2* fusion protein was generated by the insertion of a cDNA encoding *RRAS2* into the pGEX-4 T3 vector as recommended by the manufacturer (Pharmacia). Antibody binding was detected using a horseradish peroxidase-conjugated anti-rabbit immunoglobulin G (GE Healthcare UK), which was visualized by chemiluminescence (BioRad Laboratories, California, USA) after washing 3 times with 1% TBS Tween buffer and by exposure of the membrane to X-ray film.

### R-RAS2 expression

R-RAS2 expression was assessed in the commercial Brain Tumor Screen (CC17-11-004: n = 200) and Brain Medulloblastoma (CC17-11-002: n = 60) tissue microarrays (Cybrdi). The arrays were equilibrated in 0.3 M NaCl, 0.1 M Tris–HCl [pH 7.4] containing 1% (v/v) newborn calf serum and then probed at 4°C overnight with the specific anti-α-R-RAS2 antibody (1:100). After washing thoroughly, antibody binding was detected with a biotinylated donkey anti-rabbit IgG (1 h) and then with a streptavidin-biotinylated horseradish peroxidase complex (1 h). The arrays were again washed and they were then incubated for a further 6 minutes in 0.1 M sodium phosphate in the presence of H_2_O_2_ (0.1 mg/ml) and diaminobenzidine (0.5 mg/ml), producing a colorimetric reaction that was stopped by adding 0.1 M sodium phosphate.

### Sample quantification

The expression of R-RAS2 in immunohistochemical (IHC) images was evaluated by calculating the integrated density of DAB in four equal and representative regions of interest in each sample using ImageJ 1.44c analysis software [23] The integrated density calculation represents the sum of the values of the pixels in the image or selection, which is equivalent to the product of the area and the mean grayscale value. For RGB images, the mean is calculated by converting each pixel to an 8-bit grayscale using the formula gray  =  (red  +  green  +  blue) ⁄ 3. The average density measurements of the samples were compared with those obtained from the controls and the results were expressed as a percentage of the corresponding control. Each sample was assessed by 5 independent evaluators.

### RT-qPCR

#### RNA samples

A total of 32 human samples, including normal and brain cancer tissue samples, were analyzed to assess the expression of the genes of interest. The cancer tissues represented 4 different clinical stages and where possible, mixed ages, genders and ethnic groups. Total RNA was isolated from the selected tissue using Cytomyx (HBRT102, Lexington, MA) and it was subjected to stringent quality control. The high-quality total RNA obtained by Cytomyx was sent to OriGene Technologies, who generated 1st strand cDNA that was subsequently reverse transcribed using an oligo-dT primer. The complementary DNAs (cDNAs) were synthesized using a protocol optimized to generate long and rare cDNAs, and to eliminate genomic DNA contamination. These cDNAs were transferred into Tissue qPCR Array plates containing dried, PCR-ready, first-strand cDNAs (Brain Cancer cDNA Array I from OriGene Technologies).

#### qPCR

PCR was performed in intron-spanning assays using the primers *RRAS1* (ENSG00000126458; Fw 5′-GGCAGATCTGGAGTCACAGC-3′, Rv 5′-ACGTTGAGACGCAGTTTGG-3′) and *RRAS3* (ENSG00000158186; Fw 5′- CAGCTTTGAGCACGTGGA-3′, Rv 5′- CGAGGATCATCGGGAATG-3′), designed using Probe Finder software (Roche Applied Science, https://www.roche-applied-science.com/sis/rtpcr/upl/index.jsp). Specific intron-spanning primers for *RRAS2* (ENSG00000133818; Fw 5′-AGCACGGCAGCTTAAGGTAA-3′, Rv 5′-CTTTCCGTGTTGGTTCTGGT-3′) were designed using Primer Express 2.0 software (Applied Biosystems). The specificity of each assay was determined by performing a BLAST search. A non-intron spanning assay was prepared by OriGene Technologies using primers designed against the *Homo sapiens* β-actin *(ACTB)* sequence (NCBI accession # NM_001101). *RRAS1* and *RRAS3* were provided by Sigma Aldrich, *RRAS2* by Invitrogen and ACTB by OriGene Technologies.

The qPCR reactions were performed manually as indicated by OriGene, except that the lyophilized cDNA was diluted in twice the suggested volume of water (to evaluate 2 genes per plate instead of 1) and the reactions were transferred to a 384-well plate. The remaining reagents were added in the suggested proportions and qPCR was performed on an ABI 7900HT Fast Real-Time PCR System (Applied Biosystems), using Sybr green as a fluorescent dye for detection. Relative gene expression was normalized to ACTB expression according to the 2^-ΔΔCt^ method (Livak & Schmittgen, 2001), as suggested by OriGene Technologies. This approach reduced the dispersion of the expression data within the biological groups and validated this approach. The qPCR data was analyzed with GenEx Professional 5.3.7 software (MultiD, Sweden).

### Statistical analyses

All results are expressed as the mean ± standard deviation (SD). The experimental groups were compared using a two-sample Student’s t-test. To corroborate these statistical differences, we performed an additional ANOVA test, assembling the samples in groups according to tumor type. The Student’s t-test and ANOVA p values are indicated in the figure legends.

### Exclusion criteria

Samples exhibiting necrosis or more than 20% contamination with normal tissue were excluded from the analyses (C2, C9, C10, D7, D8, D9, D10, E11, E12, F4, F6 and F7 from Brain Cancer cDNA Array, sample number 178 from CC17-11-004 Array, and samples 13, 14 and 15 from CC17-01-002 Array). The tumors for which the degree of malignancy was not established were also excluded from the study (F8, F9, F10, F11 and F12 from Brain Cancer cDNA Array). Similarly, the samples whose values had a mean deviation ^+^/_−_ 2SD were also eliminated from the study (C4, C11, D1, E1 and E12 from Brain Cancer cDNA Array; samples numbers 22, 37, 38, 75, 85, 150 and 152 from CC17-01-002 Array). The remaining samples were all included in the study.

## Results

### Expression of the R-RAS2 protein in human CNS tumors

**Figure 1:**
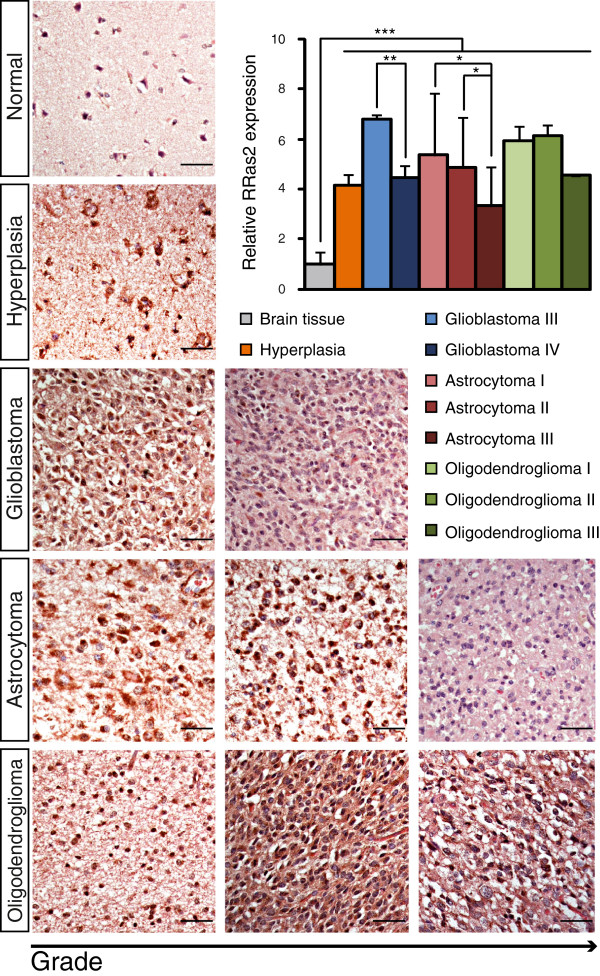
**R-RAS2 protein expression in human CNS tumors.** Samples were obtained from the commercial Brain Tumor Screen tissue microarray (CC17-11-004). Cryostat sections of human brain tissue (from normal brain, hyperplasias, glioblastoma grade III or IV, astrocytoma grades I, II or III, and oligodendroglioma grades I, II or III) were analyzed by immunostaining with antibodies against R-RAS2, and they were counterstained with hematoxylin and eosin. A quantitative analysis of R-RAS2 expression was performed for each brain tumor subtype, normalizing the R-RAS2 values to the levels expressed in normal tissue. R-RAS2 protein expression is represented as the diaminobenzidine absorbance, which reflects the presence of the protein in the samples. Student’s t-test *p < 0.05, **p < 0.0005, ***p < 0.00005. ANOVA p < 0.00001. Scale bars = 50 μm.

We analyzed the expression of R-RAS2 in 199 human CNS tumor samples from 105 patients, with a wide range of tumor types (Table 1a). The expression of the R-RAS2 protein was assessed by immunohistochemistry using the polyclonal antiserum described previously, the specificity of which was confirmed by immunoblotting total brain homogenates of wild type and *RRas2*^*−/−*^ mice (Additional file 1: Figure S1c). R-RAS2 was overexpressed in all cancer types and grades when compared to the 23 normal brain tissue samples taken from patients (Figure 1, Additional file 1: Figure S1a and Additional file 2: Figure S3). Densitometric quantification revealed R-RAS2 protein expression to be between 3- and 7-fold higher in malignant tissue (n = 170) than in normal brain tissue (n = 23), although R-RAS2 overexpression appeared to be inversely correlated with the tumor grade for all the tumor types analyzed. Thus, R-RAS2 was overexpressed approximately 8-fold more in grade III glioblastomas (n = 13) when compared to normal tissue, while only a 4-fold increase in expression was observed in grade IV tumors (n = 15: Figure 1). Likewise, R-RAS2 expression increased 5-fold in grade I-II astrocytomas but only 3-fold in grade III astrocytomas. Finally, R-RAS2 expression increased approximately 7-fold in grade I-II oligodendrogliomas (n = 9) and 4-fold in grade III tumors (n = 4). Interestingly, R-RAS2 protein overexpression was also associated with non-malignant hyperplasia in the brain (Figure 1 and Additional file 2: Figure S3). Together, these results suggest that R-RAS2 is overexpressed in a wide variety of brain tumors, yet more intensely in pre-malignant conditions. Moreover, R-RAS2 overexpression is also more pronounced in lower grade tumors than in high-grade glioblastomas, astrocytomas and oligodendrogliomas (Figure 1, Additional file 1: Figure S1a and Additional file 2: Figure S3).

**Table 1:** Samples obtained from a commercial brain

**Histo-Pathology**	**Grade**^**1**^	**Patients**	**Samples**	**Mean age**	**Men (%)**
**a) Clinicopathologic data of 193 human CNS samples**					
**Brain tissue**	-	13	23	35,3 ± 19,2	38,5
**Hyperplasia**	-	2	3	34,7 ± 8,1	50
**Mixed glioma**	-	2	4	41 ± 3,5	100
**Glioblastoma**	III	7	13	60,5 ± 14,4	85,7
	IV	9	15	49,5 ± 16,9	66,6
**Astrocytoma**	I	32	59	37,6 ± 10,3	69,6
	II	28	54	43,5 ± 14,2	60,7
	III	5	9	44,4 ± 7,3	20
**Oligodendroglioma**	I-II	2	4	29,5 ± 0,6	50
	II-III	3	5	57,4 ± 15,1	100
	III	2	4	42,5 ± 11	100
**b) Clinicopathologic data of 56 human CNS tumor samples**					
**Medulloblastoma**	IV	6	18	8,8 ± 5	66,6
**Cerebroma**	IV	3	9	26,6 ± 16,9	33,3
**Meningioma**	IV	4	11	40 ± 9,5	75
**Ependymoma**	IV	1	3	14 ± 0	100
**Undifferenciated**	IV	5	15	11,2 ± 4	60
**Histo-Pathology**	**Grade**^**1**^	**Patients**	**Mean age**	**Men (%)**	
**c) Clinicopathologic data of 32 human CNS samples**					
**Brain tissue**	-	1	55	0	
**Hyperplasia**	-	1	45 ± 0	100	
**Astrocytoma**	I	1	27 ± 0	0	
	II	2	39,5 ± 19,1	50	
	III	3	35 ± 4,6	100	
**Oligoastrocytoma**	II	1	37 ± 0	0	
	III	1	41 ± 0	100	
**Oligodendroglioma**	II	1	33 ± 0	100	
	III	3	45,3 ± 5,1	0	
**Ependymoma**	III	1	49 ±0	0	
**Meningioma**	I	13	53,8 ± 13,6	38,5	
	II	4	61 ± 11,2	50	

^1^Classified according to the WHO criteria.

**Figure 2:**
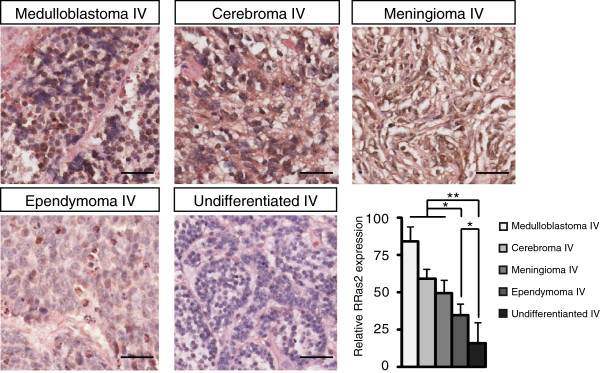
**Expression of R-RAS2 protein in tissue from highly malignant human CNS tumors.** Samples were obtained from the commercial Brain Medulloblastoma tissue microarray (CC17-01-002). Cryostat sections of human brain tumor tissue from medulloblastomas, cerebromas, meningiomas, ependymomas and undifferentiated tumor tissue were immunostained with an antiserum against R-RAS2, and counterstained with hematoxylin and eosin. R-RAS2 expression is represented as the diaminobenzidine absorbance in the samples, and R-RAS2 expression in each brain tumor subtypes was quantified and normalized to the expression in control samples Student’s t-test *p < 0.05, **p < 0.00005. ANOVA p < 0.00001. Scale bars = 50 μm.

An analysis of RRAS2 expression in a second array of highly malignant CNS tumors, 57 samples from 19 patients (Table 1b), also revealed R-RAS2 overexpression in grade IV medulloblastomas (n = 18), cerebromas (n = 9), meningioma (n = 11), ependymomas (n = 3) and undifferentiated (n = 15) tumors (Figure 2 and Additional file 1: Figure S1b). Significant differences in R-RAS2 expression were observed depending of the type and origin of the tumor, with the strongest R-RAS2 expression detected in medulloblastomas and the weakest in undifferentiated ependymoma (Figure 2).

### Overexpression of *RRAS2* mRNA in human brain tumors

**Figure 3:**
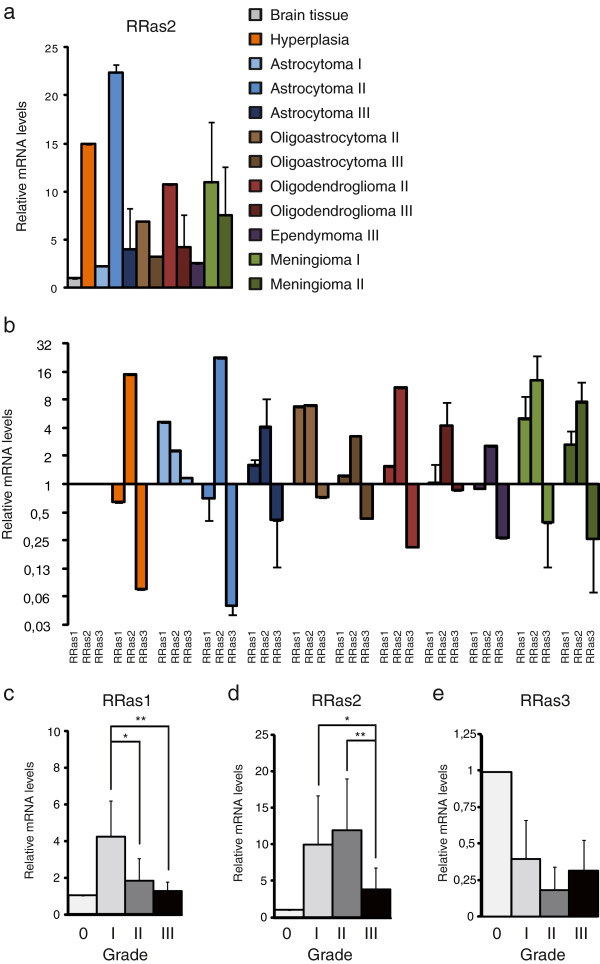
***RRAS2 *fulfils an important role in low grade tumors. a)** A qPCR analysis of the cDNA obtained from different human brain samples from the commercial Brain Cancer cDNA Array I (HBRT102) was performed using specific oligos. The mean expression of *RRAS2* mRNA is shown for each tumor subtype and grade, and all the values shown are relative to the expression of *RRAS2* mRNA in healthy tissue. **b-e)** The expression of all RRAS subfamily members is altered in tumor cells. A qPCR analysis of the cDNAs from human brain tumors represented according to the tumor type. *RRAS1*, *RRAS2* and *RRAS3* transcripts were measured in each sample from the Brain Cancer cDNA Array I (HBRT102). **c-e)** Transcript levels of the different R-RAS subfamily members were gather and analyzed according to the degree of malignancy (WHO criteria), normalizing all the data relative to the expression in healthy tissue. Student’s t-test *p < 0.05, **p < 0.01. ANOVA p < 0.05.

*RRAS2* overexpression in human CNS tumors was confirmed by RT-qPCR in tissue samples from 32 patients (Table 1c). In different brain tumor types, *RRAS2* mRNA expression was 2- to 22-fold higher than in normal tissue (Figure 3a). The highest levels of expression were found in grade II astrocytomas (22-fold increase, n = 2), whereas 4-fold increases were detected in grade III astrocytomas (n = 3), confirming that *RRAS2* is more strongly overexpressed in lower-grade tumors. *RRAS2* mRNA overexpression was also detected in pre-malignant hyperplasia (a 16-fold increase, n = 1: Figure 3a and Additional file 3: Figure S2). The only exception was a grade I astrocytoma sample in which low *RRAS2* mRNA levels were detected (n = 1), although this may have been due to misclassification. even though the number of mRNA samples classified by tumor type was not sufficiently high to generate statistically significant differences, the mRNA expression data supported the protein expression results. However, when the tumors were grouped together in function of their degree of malignancy (grade I, n = 13; grade II, n = 8; and grade III, n = 8) statistically significant differences in expression were observed (Figure 3d).

### Confirmation of R-RAS2 expression in western blots

**Figure 4:**
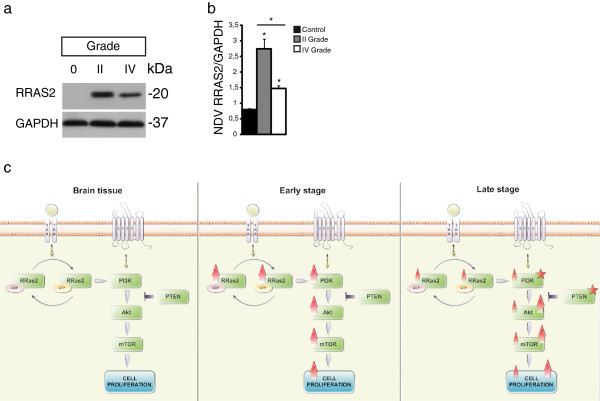
**Increased R-RAS2 expression in astrocytomas. a)** R-RAS2 and GAPDH expression in lysates of control brain tissue (n = 3), grade II astrocytoma (n = 5) and grade IV astrocytoma (n = 5) analyzed in Western blots. **b)** Graph show the mean and SD of the signal quantified relative to the amount of protein in the control, grade II and IV astrocytomas. Student’s t-test p < 0.005. **c)** Diagram showing the hypothesis of the role of R-RAS2 at different tumor stages.

We analyzed the expression of R-RAS2 in western blots of low grade (n = 5) and high grade (n = 5) malignant astrocytomas (Table 2). The results obtained indicate the existence of R-RAS2 overexpression associated with tumor progression as opposed to the control tissue (n = 3). Furthermore, stronger expression was observed in tumors with a lower degree of malignancy (Figure 4a and b). These data confirm the results obtained previously by immunohistochemistry and RT-qPCR, where R-RAS2 was seen to be expressed more strongly in the lower grade tumors as opposed to the higher grade tumors (p < 0.05; Figure 4b).

**Table 2:** Clinical data of different grade astrocytomas analyzed in western blots

**Histo-pathology**	**Grade**^**1**^	**Patients**	**Samples**	**Mean age**	**Men (%)**
**Brain tissue**	-	3	3	41,3 ± 37	33,3
**Astrocitoma**	II	5	5	44 ± 10	40
**Glioblastoma**	IV	5	5	66,6 ± 13	40

^1^Classified according to the WHO criteria.

### In CNS tumors, *R-RAS2* is the most strongly overexpressed gene within the *R-RAS* subfamily

*RRAS2* gene expression was compared by qPCR with that of the other 2 genes in this R-RAS subfamily (*RRAS1* and *RRAS3*) in the same CNS tumor samples (n = 32). We observed a general increase in *RRAS1* mRNA in CNS tumors and a decrease in *RRAS3* expression in CNS tumors (Figure 3b, Additional file 3: Figure S2 and Table 3). When we assessed *RRAS1, RRAS2* and *RRAS3* expression according to tumor type and grade (Figure 3b-d), the expression of *RRAS1* and *RRAS2*, but not that of *RRAS3*, was significantly stronger in grade I and II tumors than in grade III tumors (p < 0.01). However, while *RRAS2* overexpression in grade I tumors was 13-fold higher than in normal tissue, *RRAS1* levels only experienced a 5-fold increased (Figure 3b-e).

**Table 3:** RT-qPCR results mean fold variation for RRas1, RRas2 and RRas3

				**RRas1**		**RRas2**		**RRas3**	
**Histo-pathology**	**Grade**^**1**^	**Gender**	**Age**	**Fold variation**^**2**^	**Standard deviation**^**3**^	**Fold variation**^**2**^	**Standard deviation**^**3**^	**Fold variation**^**2**^	**Standard deviation**^**3**^
**Brain tissue**	-	Female	55	1,003	0,151	1,000	0,150	1,000	0,150
**Meningioma, fibroblastic**	I	Female	56	3,054	1,004	3,596	1,183	0,254	0,083
**Meningioma, fibroblastic**	I	Female	51	7,878	0,335	-	-	0,522	0,022
**Meningioma**	I	Male	71	2,106	0,181	19,016	1,632	1,040	0,089
**Meningioma, meningothelial**	I	Female	56	4,565	1,672	8,514	3,118	0,262	0,096
**Meningioma, secretory**	I	Female	41	1,422	0,160	25,092	2,825	0,083	0,009
**Meningioma**	I	Female	42	5,798	1,248	4,779	1,029	0,263	0,057
**Meningioma, fibroblastic**	I	Male	39	-	-	13,533	4,059	0,592	0,178
**Meningioma, fibroblastic**	I	Male	43	1,944	0,640	14,801	4,873	0,133	0,044
**Astrocytoma**	I	Female	27	4,550	0,691	2,253	0,342	-	-
**Meningioma**	I	Female	83	3,043	0,414	10,352	1,407	0,251	0,034
**Meningioma**	I	Male	68	3,027	1,174	6,131	2,378	0,202	0,078
**Meningioma**	I	Female	45	6,406	5,394	2,441	2,056	0,526	0,443
**Meningioma**	I	Female	61	4,791	0,024	8,719	0,043	0,352	0,002
**Meningioma**	I	Male	44	5,386	0,485	10,301	0,927	0,688	0,062
**Meningioma, atypical**	II	Male	67	2,365	0,154	14,736	0,962	0,103	0,007
**Hyperplasia**	II	Male	45	0,636	0,043	14,934	1,004	0,075	0,005
**Oligoastrocytoma**	II	Female	37	-	-	6,902	3,282	-	-
**Astrocytoma**	II	Male	26	0,503	0,156	21,775	6,753	0,058	0,018
**Meningioma, meningothelial**	II	Female	56	2,388	1,323	4,122	2,284	0,312	0,173
**Astrocytoma**	II	Female	53	0,928	0,287	22,939	7,099	0,042	0,013
**Meningioma, atypical**	II	Male	73	1,757	1,006	7,157	4,096	0,129	0,074
**Meningioma, atypical**	II	Female	48	4,151	1,897	4,118	1,882	0,512	0,234
**Oligodendroglioma**	II	Male	33	1,545	0,269	10,723	1,868	0,211	0,037
**Astrocytoma, anaplastic**	III	Male	36	1,768	0,583	2,815	0,929	0,403	0,133
**Astrocytoma, anaplastic**	III	Male	39	1,290	0,236	0,559	0,102	0,702	0,128
**Astrocytoma**	III	Male	30	1,721	0,875	8,782	4,464	0,128	0,065
**Oligodendroglioma, anaplastic**	III	Female	41	0,814	0,185	0,579	0,131	-	-
**Ependymoma of brain, anaplastic**	III	Female	49	0,895	0,055	2,548	0,155	0,266	0,016
**Oligoastrocytoma, anaplastic**	III	Male	41	1,211	0,175	3,254	0,470	0,432	0,062
**Oligodendroglioma, anaplastic**	III	Female	51	0,521	0,075	5,038	0,721	0,165	0,024
**Oligodendroglioma, anaplastic**	III	Female	44	1,707	0,509	7,080	2,110	0,119	0,035

^1^Classified according to the WHO criteria.

^2^All results were calculated as the mean of the three measurements performed on each sample and subsequently normalized to the expression in the non tumoral sample.

^3^Standard deviation of the three measurements performed on each sample.

## Discussion

This is the first study to demonstrate that R-RAS2 is overexpressed in a wide variety of human CNS tumors. R-RAS2 was overexpressed in all the tumors analyzed when compared with normal tissue, and significantly stronger expression was detected in low-grade as opposed to high-grade tumors. Hence, R-RAS2 overexpression seems to be associated with the early stages of transformation and indeed, R-RAS2 overexpression was also associated with gliosis and hyperplasia, both of which are considered to be pre-malignant processes. When the expression of mRNA encoding the other 2 members of the RAS-related subfamily was assessed, the brain specific *RRAS3* was not overexpressed in CNS tumors. By contrast, *RRAS1* mRNA expression was enhanced in CNS tumors, albeit not to the same extent as *RRAS2*. Given that unlike *RRAS1* or *RRAS3*, *RRAS2* can transform NIH-3 T3 cells [8-11], and in light of the low frequency of oncogenic mutations in classic Ras genes, we hypothesize that R-RAS2 overexpression is an important event in the transformation of neural cells, particularly those that do not carry oncogenic RAS mutations (database reference). Nevertheless, further studies will be necessary to demonstrate this assumption.

There is compelling evidence that R-RAS2 activity is linked to the activation of the PI3K pathway. Indeed, R-RAS2 directly recruits and activates several catalytic subunits of type I PI3K [23,24]. In conjunction, these data suggest that by activating the PI3K pathway, R-RAS2 can promote neural cell survival and proliferation in association with transformation in several types of CNS cancers. This hypothesis is consistent with the high frequency of inactivating or deleting mutations in PTEN, a negative regulator of PI3K activity, in brain tumors [25-27].

One of the principal causes of cancer are increases in PTEN-PI3K signaling [28] given that mutations in PI3K or PTEN that maintain these proteins active induce tumorigenic processes [29,30]. In the CNS in particular, PTEN and PI3Kα appear among the 15 genes most frequently mutated in tumors (Cosmic database, Sanger Institute), irrespective of their location and histological type. Nevertheless, the frequency with which mutations appear in PTEN/PI3K does increase in function of the malignancy of the tumor. Thus, for example, the frequency of mutations in PTEN increases from 1% in grade I astrocytomas to 21% in grade IV astrocytomas. Likewise, PI3K does not appear to be mutated in low grade astrocytomas but the frequency of mutations in high grade malignant tumors of this type reaches 6%. The same occurs in oligodendrogliomas in which the frequency of mutations in PTEN and PI3Kα is 3-2% in low grade tumors, reaching 4 and 10%, respectively, in high grade tumors (Cosmic database, Sanger Institute).

Alternatively, activating mutations and overexpression of classical Ras subfamily members have been described as elements responsible for the development of tumors in human [3], although only 0.5% of CNS tumors present mutations in classical RAS. Indeed, mutations in *RRAS2* have not been found in the CNS (n = 7,378: Cosmic database, Sanger Institute). Given the above, we propose that the overexpression of R-RAS2 may be an important event in the initiation of the neural transformation process in low grade CNS tumors, in which the PTEN/PI3K pathway has still to be activated due to mutation.

## Conclusions

Our findings demonstrate that R-RAS2 mRNA and protein is overexpressed in a wide variety of human CNS tumors, including glioblastomas, astrocytomas, oligodendrogliomas and medulloblastomas. While R-RAS2 was overexpressed in all CNS tumor types, its expression was inversely related to the degree of malignancy, suggesting that R-RAS2 overexpression is an early event in neural cell transformation.

### Ethical standards

This study was performed in compliance with all applicable European and Spanish law.

## Competing interests

The authors declare that they have no competing interests.

## Authors’ contributions

SGE, PHV, MFA, SHG, SGF and AMR performed the experiments. BC conceived the idea, designed the experiment and drafted the manuscript. MF and BC edited the manuscript, which all the authors read and approved.

## Supplementary Material

Additional file 1: Figure S1 **a) Combined R-RAS2 expression levels in all samples from a brain tumor tissue microarray.** R-RAS2 protein levels were expressed as the diaminobenzidine absorbance that reflects the presence of the protein in the samples. The R-RAS2 in human brain tissue is expressed relative to that found in healthy brain samples. All values were normalized to the levels detected in control tissue and the error bars are due to technical errors. All samples were derived from different human CNS tumors obtained from a commercial array (CC17-11-004). **b) Combined R-RAS2 expression in samples from a brain tissue microarray.** R-RAS2 protein levels expressed as the diaminobenzidine absorbance that reflects the presence of the protein in the samples. All samples were derived from different grade IV human CNS tumors obtained from a commercial brain medulloblastoma TMA (CC17-01-002). The error bars are due to technical errors. **c) Confirmation of anti-R-RAS2 specificity.** In Western blots the antiserum raised against R-Ras2 identified the protein in total brain samples but not in lysates from mutant *RRas2*^*−/−*^ mice. As a loading control, the same aliquots were probed with anti-α tubulin antibodies (bottom). Click here for file

Additional file 2: Figure S3 R-RAS2 protein expression in different grades of tumors. Representative photographs of each tumor from the Brain Tumor Screen Tissue microArray (CC17-11-004) after the array was analyzed by immunostaining with anti-R-RAS2, and counterstained with hematoxylin and eosin. LA, Little Astrocytoma; HG, Hyperplasia of gliocyte; A, Astrocytoma; and O, Oligodendroglioma. Click here for file

Additional file 3: Figure S2 Combined results of the qPCR analyses. The cDNAs assayed were derived from different human brain samples obtained from a commercial cancer cDNA array (HBRT102). The samples were analyzed using specific oligos for *RRAS1*, *RRAS2* and *RRAS3* mRNA. *RRAS1* (a), *RRAS2* (b) and *RRAS3* (c) mRNA expression in tumor tissues and hyperplastic samples was normalized to β-actin expression, and the data are expressed relative to the mRNA expression in healthy tissue. The error bars are due to technical errors. Click here for file
