# Supplementary figures and images for: R-RAS2 overexpression in tumors of the human central nervous system
Source: Mol Cancer. 2013 Oct 23;12:127. doi: 10.1186/1476-4598-12-127 (PMC3900289; doi:10.1186/1476-4598-12-127)

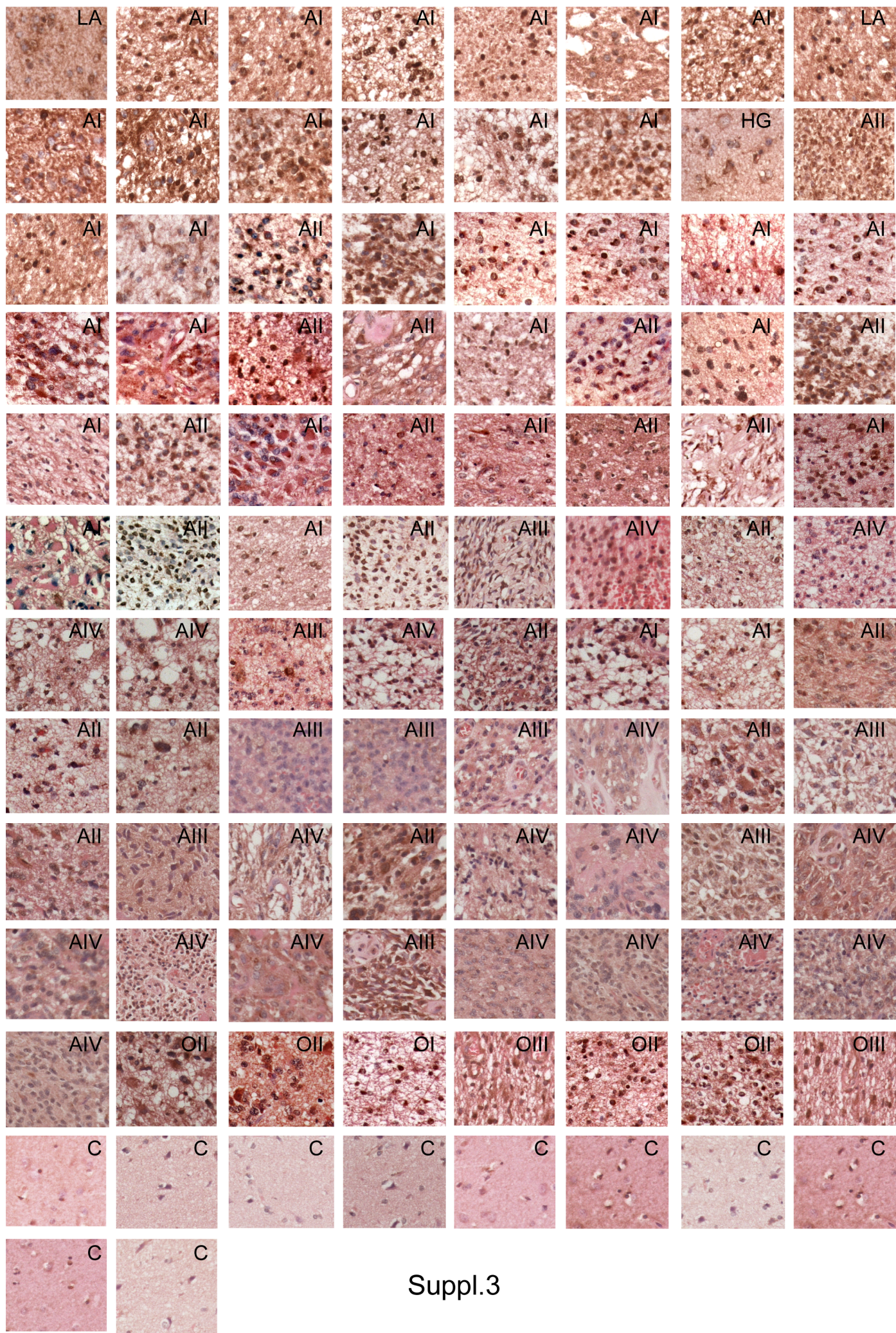

Supplement: Additional file 2: Figure S3 — R-RAS2 protein expression in different grades of tumors. Representative photographs of each tumor from the Brain Tumor Screen Tissue microArray (CC17-11-004) after the array was analyzed by immunostaining with anti-R-RAS2, and counterstained with hematoxylin and eosin. LA, Little Astrocytoma; HG, Hyperplasia of gliocyte; A, Astrocytoma; and O, Oligodendroglioma. [file 1476-4598-12-127-S2.pdf]
